# An evaluation of managed access agreements in England based on stakeholder experience

**DOI:** 10.1017/S0266462323000478

**Published:** 2023-07-27

**Authors:** Caroline Farmer, Maxwell S. Barnish, Laura A. Trigg, Samuel Hayward, Naomi Shaw, Louise Crathorne, Thomas Strong, Brad Groves, John Spoors, G. J. Melendez Torres

**Affiliations:** 1 Peninsula Technology Assessment Group (PenTAG), Department of Public Health and Sport Sciences, University of Exeter Medical School, Exeter, UK; 2 Health and Care Public Health Team, North Somerset Council; 3 Managed Access Team, National Institute for Health and Care Excellence (NICE), London, UK; 4 Medicines Value and Access Unit, NHS England, London, UK

**Keywords:** policy, reimbursement mechanisms, therapies, investigational, value-based health care

## Abstract

**Objectives:**

The objective of this research was to evaluate managed access policy in England, drawing upon the expertise of a range of stakeholders involved in its implementation.

**Methods:**

Seven focus groups were conducted with payer and health technology assessment representatives, clinicians, and representatives from industry and patient/carer organizations within England. Transcripts were analyzed using framework analysis to identify stakeholders’ views on the successes and challenges of managed access policy.

**Results:**

Stakeholders discussed the many aims of managed access within the National Health Service in England, and how competing aims had affected decision making. While stakeholders highlighted a number of priorities within eligibility criteria for managed access agreements (MAAs), stakeholders agreed that strict eligibility criteria would be challenging to implement due to the highly variable nature of innovative technologies and their indications. Participants highlighted challenges faced with implementing MAAs, including evidence generation, supporting patients during and after the end of MAAs, and agreeing and reinforcing contractual agreements with industry.

**Conclusions:**

Managed access is one strategy that can be used by payers to resolve uncertainty for innovative technologies that present challenges for reimbursement and can also deliver earlier access to promising technologies for patients. However, participants cautioned that managed access is not a “silver bullet,” and there is a need for greater clarity about the aims of managed access and how these should be prioritized in decision making. Discussions between key stakeholders involved in managed access identified challenges with implementing MAAs and these experiences should be used to inform future managed access policy.

## Introduction

Managed access is known internationally by multiple terms, including managed entry agreements [MEAs], performance-based risk-sharing schemes, or coverage with evidence generation. These approaches aim to deliver on policy goals to ensure earlier patient access to promising technologies with an uncertain evidence base, while supporting innovation in a way that facilitates a more robust and holistic assessment of the technology for reimbursement. Managed access in England is used to provide patients with time-limited access to the most promising new medicines that are unable to be recommended for routine use, while further evidence is collected to prove they are a good use of National Health Service (NHS) resources. Evaluations of managed access policies to date conclude that there are both advantages and limitations, with advantages largely determined by the way in which the policy is implemented (1–4). Key advantages of managed access include patients receiving access to technologies they may not have been able to otherwise, industry is supported with data collection within the target health services, and the health system ensures that technologies that reach patients through routine commissioning are both clinically and cost-effective for patients. However, there remain concerns over the substantial administration and expense of managed access agreements (MAAs) for the health system, whether evidence generation completed during the MAA is of sufficient quality to further support decision making, and whether spending on MAAs diverts funding away from other innovative products such as surgical and medical device technologies (4–8).

In England, the potential for managed access has recently expanded. To date, the vast majority of MAAs have been implemented for cancer indications via the Cancer Drugs Fund (CDF), but in 2022 the UK government launched the Innovative Medicines Fund (IMF), which matches the level of funding available for cancer MAA (£340m [$406m] per year) with new funding for noncancer topics (£340m [$406m] per year) to create a total funding allocation of £680m [$812m] per year. This decision is consistent with the UK’s policy priorities for the NHS, including the NHS Long-Term Plan (9), the Rare Disease Framework (10), and the UK Life Science Vision (11). The National Institute for Health and Care Excellence (NICE) has made fifty-two Cancer Drugs Fund recommendations and twenty-five have had guidance updated following a period of managed access, 92 percent recommended for routine funding (12). However, due to the confidential nature of commercial negotiations and patient information, key information (including the final prices for technologies paid by the healthcare system) is rarely available in the public domain. Several reviews of managed access policy have been published (2;3;7;8;13;14), which have attempted to evaluate the success of managed access through metrics available in the public domain, such as the characteristics of technologies at entry (e.g., clinical efficacy data) and total expenditure for the policy. These reviews provide many learnings to policy makers about the way managed access policies have been designed and some of the common barriers and pitfalls. However, it is challenging for reviews to capture the way in which managed access policy has changed over time, both iterative changes as well as large-scale reform, such as that to the UK Cancer Drugs fund in 2016. Generalized metrics of the performance of managed access are also unable to account for significant heterogeneity across indications and technologies included in MAAs. For example, aggregate data for a common clinical metric (e.g., overall survival) may not capture the broad range of anticipated clinical value that eligible innovative technologies offer, or the maturity of clinical evidence at the time of submission. It is therefore crucial that any evaluations related to the performance of managed access take into consideration the clinical and financial data within the context of the appraisal.

An evaluation of managed access was conducted in England by drawing upon the experiences of key stakeholder groups involved in the implementation of managed access policy. Stakeholders are able to provide an in-depth insight into the implementation of managed access that draws upon their experience and knowledge of the appraisal context, and without requiring that commercially sensitive information be shared. This, therefore, offers a significant advantage to reviews conducted by third parties. The results of this evaluation were intended to provide a comprehensive appraisal of managed access in England and suggest key areas for improvement within the context of the IMF.

## Methods

### Study Design

A qualitative focus group methodology (15) was used to investigate stakeholders’ views on managed access. Qualitative framework analysis (16) was used as the analytical method.

### Participants

Seven focus groups were conducted with representatives from a single stakeholder group. Purposive sampling was employed to select UK-based representatives who had involvement or responsibility for existing MAAs. Stakeholders were selected to cover a full range of perspectives on managed access in England. Lead contacts at each organization were responsible for selecting representatives, in discussion with the project lead. The following stakeholders were involved: (i) payers, (ii) health technology assessment (HTA) representatives, (iii) UK government officials, (iv) clinical experts, (v) patient group representatives, and (vi) industry trade body representatives.

### Focus Group Conduct

The project team coordinated the schedule with lead contacts at each stakeholder organization. The semi-structured topic guide was developed by the evaluators and agreed following consultation with the project leads. The topic guide was based on four research questions: (i) How successful have MAAs been in delivering on their objectives? (ii) What are the common problems experienced during MAAs? (iii) What characteristics define appraisals that should be considered for a MAA? and (iv) What are the common reasons why a potentially relevant MAA does not go ahead? Questions were adjusted for each stakeholder group, both to target the expertise of the particular group, and to reflect the researchers’ evolving understanding of managed access through reflexive practice (17). An example topic guide is provided in Supplementary File 1.

Focus groups took place in February and March 2021 and lasted 45–60 min. Sessions were conducted online using videoconferencing. Sessions were chaired by the lead author. The third author was in attendance to take notes to inform the focus group schedule. The chat function was used during sessions with text contributions added to the analysis. One participant, an industry representative, was unwell and unable to attend their respective focus group and was invited to submit a contribution to the focus group schedule by email.

### Data Analysis

Recordings from the focus groups were transcribed by an independent professional transcriber. Once transcripts were reviewed by two independent members of the research team as an accurate record of the focus groups, audio recordings were deleted as required by the Ethics committee. Transcripts were initially coded following a qualitative framework analysis method (16) by the first author and second coded for validation by the second author. The initial frame was based on previous appraisals of MAAs (both nationally and internationally) (2;3;6–8;18;19), and through discussion with NHS England (NHSE) and NICE about key areas of interest in development of the IMF (eighth and ninth authors). Coding was conducted over several levels, with codes at each level validated by another member of the research team. Codes were categorized within the frame and iteratively refined into themes and subthemes. Themes were discussed within the research team and all members agreed upon the final themes and subthemes.

### Ethical Approval

The project was approved by the appropriate ethics committee at the lead author’s institution.

## Results

In total, fifty-seven individuals participated in the seven focus groups: six payer representatives; seven HTA body representatives; nine HTA Committee Chairs (including other senior Committee members such as Vice-Chairs); seven government officials; eleven patient representatives; eight clinicians; and nine industry representatives. Demographic data for participants were not collected for ethical reasons, as these data were not considered necessary to address the research questions. All participants had direct experience with one or more MAA, and/or were involved in developing policy related to managed access.

Four major themes emerged from the analysis: understanding the aims of managed access; measuring the success of managed access; challenges of managed access; and eligibility for managed access (see Table 1). No themes arose from research question (iv) about why a potentially relevant MAA did not go ahead, as stakeholders could not identify any instance of this. Example quotes supporting each of the themes are provided in Supplementary File 1.

**Table 1.** Focus group themes: Evaluating the success of managed access agreements in England

Theme	Summary	Subthemes
Understanding the aims of MA	Perceptions on the aims of MAAs to date Appropriateness and compatibility of aims	–
Measuring the success of MA	Perceptions of the success of MA	Decision making Evidence generation Policy objectives Commercial objectives Finance Delivering early access Patient care
Challenges of MA	Practical challenges of MA	Delays Evidence generation Stakeholder engagement Changes in context Contracting
Eligibility for MA	Priorities for future MAAs	Determining the scope of MA Impact Risk management Broader context

MAA, managed access agreement.

### Understanding the Aims of Managed Access

**Figure 1.**
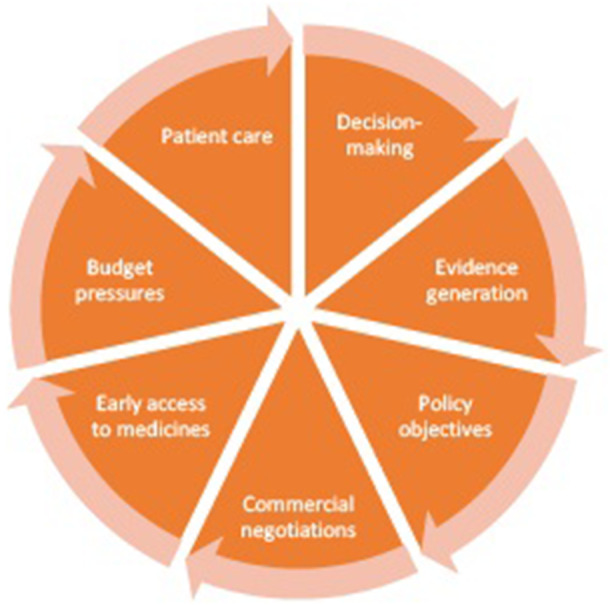
The aims of managed access agreements in England.

Stakeholders considered that a change in the concept and use of managed access over time had introduced a lack of clarity about the current priorities for its use. The CDF was originally introduced in 2010 as part of government policy to increase access to novel oncology products but was reformed in 2016 because of financial and operational pressures (2;5). Stakeholders considered that the reform has introduced a broader spectrum of aims (see Figure 1), which may be unrealistic (managed access is not a “silver bullet,” NICE Committee Chair). Stakeholders also considered that communication about the aims of managed access had been unclear, including a lack of guidance about where the priorities for managed access lie where there are conflicts between aims (e.g., where a technology entering a MAA would achieve a policy goal), but there are concerns about budget or the feasibility of evidence generation. In practice, stakeholders considered that a lack of clarity about the aims for managed access has led to inconsistency in decision making across appraisals. Some stakeholders also felt there had been instances where decisions at reappraisal appeared to prioritize different aims than earlier appraisals of the same technology, such as prioritizing budgetary concerns more or less compared to other aims. While this may be a consequence of a change in the available evidence, it had created concerns about inconsistency in decision making. Stakeholders considered that improving messaging about the aims of managed access, and their relative importance would have benefits for consistency and transparency of decision making in managed access.

### How Successful Has Managed Access Been in Achieving Its Objectives?

#### Evidence Generation

Stakeholders considered the success of evidence generation within managed access to be a key indicator of success. Evidence generation was considered successful when it reduced uncertainties highlighted by the NICE committee, and thus could be used to narrow the uncertainty in decision making. Some stakeholders discussed specific MAAs where the evidence collected, either through the maturation of clinical trial data or real-world evidence (RWE) collected in NHS services, had been instrumental in informing NICE committee decision making. However, stakeholders raised concerns that the evidence generated within MAAs could be considered relatively poor quality compared to clinical research in other areas. This was due to known issues with RWE including bias and confounding, and the length of MAAs, which puts significant pressure on the preparation and analysis of RWE. Quality issues with evidence generation limited stakeholders’ confidence in using these data for decision making. While RWE was considered to be useful where this was consistent with trial data, thus reducing uncertainty in trial outcomes, NICE committees may be more unsure how to proceed where outcomes from RWE and clinical trials varied. In this scenario, without further data collection, it may not be possible to determine which is the more plausible finding, particularly if there are known issues with the external validity of the trial evidence. Stakeholders further noted that while patient- and carer-reported outcomes were often crucial to understanding the outcomes of treatments, these may best be collected outside of a MAA. This is because stakeholders were concerned that continued access to treatment following the MAA may confound reporting and put patients/carers under pressure to “perform” (i.e., report positive and downplay negative outcomes and experiences). Finally, while stakeholders noted that RWE may be important to informing key assumptions in cost-effectiveness evaluation, it was considered that these outcomes alone may be more efficiently captured by studies separate to a MAA.

#### Decision Making

Managed access is a policy intended to aid decision making on reimbursement, however, stakeholders proposed that in some cases, a MAA had been used as a way of delaying or avoiding negative decisions, even where further evidence generation was expected to have limited value for decision making. These instances were typically for transformative medicines in areas of high unmet need where there was deadlock between industry and the healthcare system about the cost-effectiveness of the company’s proposed value proposition and MAAs offered a solution to avoid a negative recommendation.

Decisions at the end of managed access may also be particularly challenging for noncancer indications, where these historically consider the withdrawal of an available treatment, which may disincentivize a negative decision. While NICE committee chairs stated that this did not bias their decision making, NICE committees are nevertheless aware that a negative decision may involve the withdrawal of treatment from patients treated during the MAA, and will involve the restructure of health services that have adapted to deliver the new treatment. Moreover, some stakeholders were concerned that the potential for a disincentive at reappraisal would cause industry to be less flexible in commercial negotiations at the end of the MAA. MAAs in the Cancer Drugs Fund require companies to commit to funding ongoing treatment for patients treated during the managed access period in the event of a negative recommendation from NICE at the end of the MAA, this approach is also confirmed within the IMF. This policy remains unpopular amongst industry stakeholders, particularly for life-long conditions, due to the commercial implications, but other stakeholders considered that this approach reduces pressure on decision making and reduces uncertainty for patients treated within managed access.

#### Policy Objectives

Managed access was considered by stakeholders to be a relatively popular policy as it reduces the number of negative recommendations for health technologies and, in the event of a negative recommendation, it showed that NICE had engaged with industry to fully explore a route that would allow for patient access. Several stakeholders also noted that managed access showcases the UK as an investor in innovative technologies and that accelerating earlier patient access was consistent with overarching policy objectives. The utilization of MAA to achieve pure policy goals was considered to be controversial by stakeholders – whilst the mechanism was considered an important tool in a suite of commercial options available to companies – it is the research aim and the ability to resolve decision uncertainty which should always take precedent.

#### Commercial Objectives

Stakeholders considered that managed access had facilitated more candid conversations between NHSE and industry about pricing and had allowed for a more flexible approach to commercial negotiations (20), beyond Patient Access Schemes (PAS). These benefits were thought to have facilitated access to several technologies where a recommendation for routine commissioning was not possible due to significant uncertainties. Stakeholders suggested that managed access had resulted in the NHS achieving better value for some technologies, since the uncertainty is expected to be mitigated through an additional commercial agreement during the period of managed access. Risk is also shared between the company and the healthcare system during the managed access period, at which point further evidence is used to inform the decision whether to make the technology available routinely.

Conversely, some stakeholders raised concerns that managed access had, to some extent, offered an attractive alternative to routine commissioning, as companies may prefer to enter a MAA than negotiate a simple discount, such as through a PAS. Industry stakeholders stated that managed access could be viewed as a less attractive option since the introduction of additional pricing and exit requirements (e.g., funding of ongoing treatment) and that debates over these requirements had impacted negotiations for MAAs.

#### Financial

At the time of the focus groups, participants noted that few MAAs have yet reached completion, particularly for noncancer indications, and therefore the real impact of managed access on NHS spending was still to be determined. Based on their own experience of MAAs, stakeholders had mixed views about whether managed access has delivered an overall financial benefit for the NHS. Despite commercial approaches to share the risk surrounding uncertainty, managed access was still challenging due to the unclear clinical and cost-effectiveness of technologies. This uncertainty caused some stakeholders to question whether the opportunity costs of managed access could be justified, since some technologies would ultimately lead to a negative recommendation. It was noted that, particularly for noncancer indications, managed access can be expensive and time-consuming to administer for the NHS and industry, particularly in terms of evidence generation, where there can be a significant burden for staff collecting and analyzing RWE. Concerns were also raised that the costs of MAAs mean that the NHS is “paying twice” for the uncertainty in the evidence base, as MAAs are also often accompanied with higher willingness to pay thresholds (e.g., those meet criteria for the NICE Highly Specialised Technology [HST] program). Stakeholders considered that further analysis of the financial impact versus the costs of managed access was needed once more MAAs had exited the NICE process.

#### Delivering Early Access

Participants agreed that managed access has been successful in delivering earlier patient access to technologies. Not least because it had enabled patient access to treatments which might not have otherwise been recommended, due to significant evidential uncertainty. The benefits of earlier access to technologies were described as “transformative” for the lives of many patients. Managed access, and related schemes such as accelerated regulatory processes, were considered essential in a landscape requiring ever earlier access to technologies. Participants acknowledged the benefits of early access but were also concerned about how pressure for early access affected the other objectives of managed access. This included concerns about the risks of early access for patient wellbeing, commercial negotiations, and NHS budgets. There were also equity concerns about access delivered within the context of managed access, where eligibility for treatment may be narrower than the licensed population.

#### Delivering Patient Care

Enabling patient access to promising new treatments was considered one of the key objectives of managed access, and stakeholders considered that managed access would deliver overall benefits for patients. However, stakeholders discussed considerations for managed access in delivering patient care. Participants highlighted a number of ways in which MAAs may have a negative impact on patients and carers (Table 2) and discussed how these issues should not be forgotten in pursuit of the broader benefits surrounding access. Stakeholders considered it was important for the NHS and industry to communicate with patients and carers affected by a MAA, including providing regular updates during the timeline and providing clear information about what would happen at the end of the agreement. Finally, stakeholders also noted that managed access has opportunity costs for patients elsewhere in the health care system and felt that managed access should be used sparingly and appropriately to ensure that these were always justified.

**Table 2.** Potential negative impacts of MAAs for patients and carers

Risks to health of treating patients with technologies not yet proven to be clinically effective Patients and carers may experience anxiety throughout the MA period about continued access to the technology If a decision is made to withdraw a technology, this can have devastating impacts for those directly and indirectly affected The burden of data collection for patients and carers, particularly where additional assessments regularly require travel The psychological burden for patients and carers of knowing that continued access to the technology depends on their “performance” during assessments The legal paperwork required for MA may be stressful Growing use of MAAs can exacerbate inequity where agreements use selective eligibility criteria There are concerns about the ethics of delivering and measuring care within MAAs, which are not subject to the same ethical requirements as clinical trials The lack of transparency due to the commercial sensitivities of MA leave patients feeling “shut out,” and patient group reps feel uncertain how to support their members

MAA, managed access agreement.

### Challenges in Managed Access

**Figure 2.**
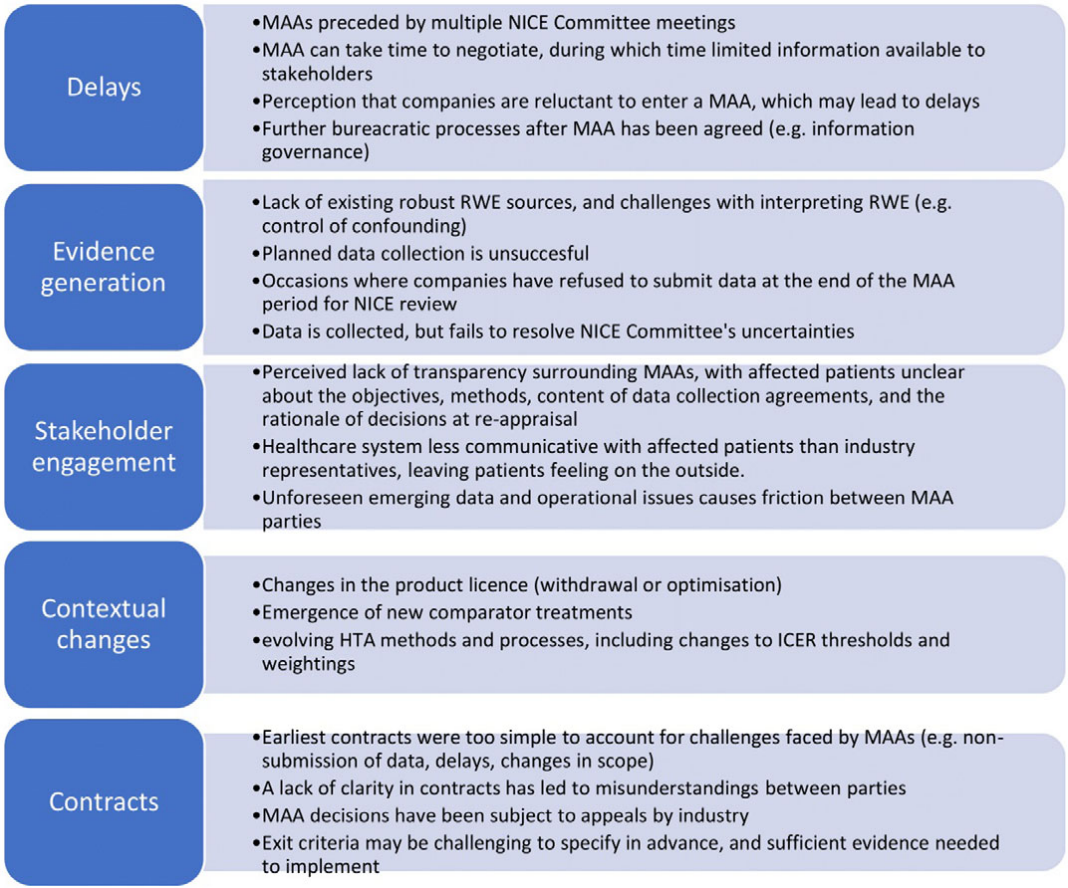
Challenges faced by MAAs since their inception. HTA, health technology assessment; ICER, incremental cost-effectiveness ratio; MAA, managed access agreement; NICE, National Institute for Health and Care Excellence; RWE, real-world evidence.

Stakeholders discussed a number of common challenges associated with existing MAAs. These challenges are outlined in Figure 2.

### Future Eligibility for Managed Access

**Figure 3.**
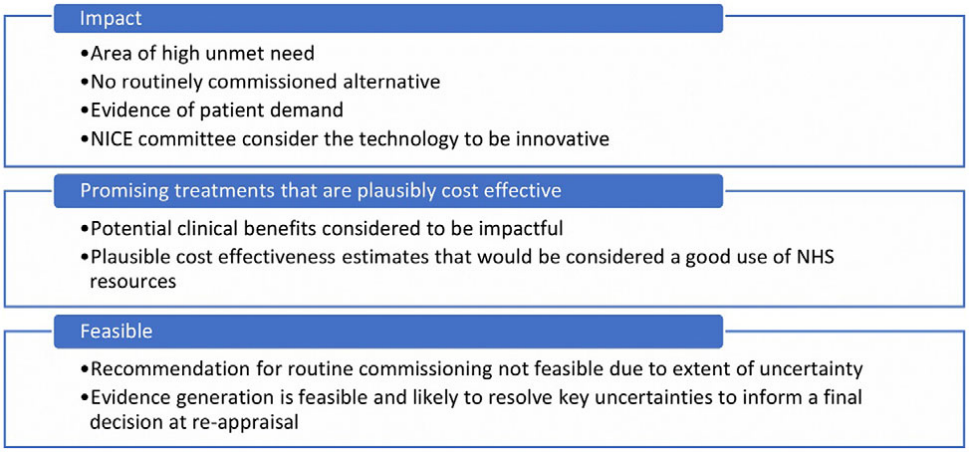
Key eligibility criteria for technologies to enter MA discussed by stakeholders. NICE, National Institute for Health and Care Excellence.

Few stakeholders considered that the eligibility criteria for managed access should be broadened (e.g., to include technologies not considered innovative), or to reappraise technologies based on new evidence generated after they have entered routine commissioning. Overall, stakeholders felt that the resource needs and risk of managed access required it to be limited to a subset of technologies where the benefit of managed access was greatest. For many technologies, stakeholders considered that routine commissioning with a simple PAS discount was achievable and would account for uncertainties in effectiveness while providing more certainty to patients. Key eligibility criteria for managed access proposed by stakeholders are shown in Figure 3.

## Discussion

This study evaluated the aims and effectiveness of the managed access approach in England by drawing on the expertise of a wide range of stakeholders directly involved in MAAs. Overall, there was strong support for the use of MAAs as a tool to facilitate decision making for innovative technologies that present challenges for typical commissioning routes due to significant evidential uncertainty. In particular, MAAs may facilitate evidence generation in the target patient population within NHS settings, thus increasing earlier access to technologies for patients with high unmet needs. MAAs can also be accompanied by commercial tools tailored to the uncertainties in the evidence, thus reducing the risk for NHS budgets. As a policy, managed access appeals to patients, particularly where they may otherwise experience delays in accessing treatments due to deadlock in decision making between companies and payers.

Stakeholders also considered that managed access was often viewed as a “silver bullet” to address all scenarios where a decision about routine commissioning was not possible due to a wide range of potential issues, beyond evidential uncertainty. Participants considered that where managed access might be used as a solution to wider issues, this increased the risks to healthcare spending and patients. In these circumstances, there are alternative reimbursement models that payers may consider (21). Due to the variability of technologies likely to benefit from managed access, it is not possible to define rigid eligibility criteria, though criteria discussed by participants broadly correspond with societal preferences (22;23) and those outlined for the IMF (24). Participants considered that payers should clearly define the aims for managed access in their healthcare systems, including guidance on how decisions will be made where these create conflict between policy aims. Most typically, this would involve situations where technologies offer transformative health benefits in an area of significant unmet need, but the value proposition from industry does not meet payer criteria.

Discussions with stakeholders highlighted several challenges with implementing MAAs, the resolution of which would increase the effectiveness of the policy. These include earlier engagement between stakeholders and streamlining the process between licensing, HTA appraisal, and entry to managed access to reduce delays to decision making. Investment in mechanisms to enhance RWE collection within NHS settings, both clinical and financial, may be needed to increase the quality and value of RWE generation within MAAs. This could include greater financial and methodological support for disease registries, and the development of a shared, consistent method for recording financial data across hospital trusts. Ensuring that there is sufficient evidence at reappraisal to inform decision making is a key metric of managed access, as there is no scope to extent the data collection period and, to date, no formalized process to reappraise treatments that have been reimbursed through managed access. In addition to supporting decision making at reappraisal, improvements in RWE generation would have broader benefits across HTA (3) and align with the UK real-world evidence framework (25). Stakeholder engagement is crucial for implementing MAAs, with clear communication between payers, companies, and patients delivering benefits for all. Discussions between payers and companies should explicitly consider the division of benefits and risk associated with the MAA and set clear expectations for the responsibilities of each party for generating evidence. An ongoing area of difficulty surrounds contracting for MAAs, since a standard contract will not account for the diversity in health conditions and technologies. Exit criteria need to be considered up-front with clear mitigation in place. One-off advanced medicinal products (ATMPs) add additional complications to exit strategies for the payer, most notably when there is a dynamic care pathway, and where the epidemiological balance is heavily weighted to the prevalent patient population. Patients and carers should also be given a clear plan for transition in the event of exit from the MAA. Patients are excluded from much of the discussions about commercial arrangements, due to confidential clinical and financial data, however, they experience a significant psychological burden throughout due to the requirements for evidence generation and the uncertainty about how the final decision will impact their continuing access to treatment. Patients, and their carers, should therefore be able to access clear information about the process involved in managed access and should be kept informed throughout the appraisal timeline.

### Strengths and Limitations

One of the key metrics of success for MAAs would be the extent to which they deliver health gains and financial benefits to the NHS compared to the alternative (i.e., technologies either not recommended through routine commissioning, or else recommended at a price different to that reached at the end of the MAA). These data were not yet available at the time of this study, though it is unlikely that such data would be available for publication due to the commercial sensitivity of this information. A strength of this research is that the expertise of stakeholders directly involved in managed access can provide an evaluation of the successes and challenges of the policy without requiring access to commercially sensitive data. Nevertheless, while the findings were based on a diverse group of stakeholders from varying perspectives in the managed access process, this was a UK-based study and views may not encapsulate international expertise with managed access across diverse healthcare settings. Finally, due to time constraints, it was not possible to delve into stakeholder experience with individual MAAs in any depth, and stakeholders typically provided a view across their experiences.

## Conclusion

This study has provided key insight into the advantages and challenges of managed access experienced by a range of stakeholders. The operational delivery of the IMF and future MAAs should address many of the considerations highlighted by stakeholders to better mitigate risks and limitations of the approach to managed access to date.

## Supporting information

Farmer et al. supplementary material Farmer et al. supplementary material

## Supplementary material

The supplementary material for this article can be found at https://doi.org/10.1017/S0266462323000478.
