# Supplementary material for: An evaluation of managed access agreements in England based on stakeholder experience
Source: Int J Technol Assess Health Care. 2023 Jul 27;39(1):e55. doi: 10.1017/S0266462323000478 (PMC11569988; doi:10.1017/S0266462323000478)
Supplement: Farmer et al. supplementary material [file S0266462323000478sup001.docx]

# **SUPPLEMENTARY FILE 1**

## Example topic guide

The following topic guide was used in the workshop with clinicians, which was the penultimate focus group. As compared to earlier focus groups, this schedule did not include discussion of research question iv (why a potentially relevant MAA does not go ahead) following feedback from other participants that this was not a key issue. Time was allowed to discuss the implications of ending MAAs, following discussions about the role of exit criteria in MAAs and the impact of withdrawing treatments from patients and health services. Timings specified were approximate and allowed time for further discussion of these and new topics within the 60-minute session.


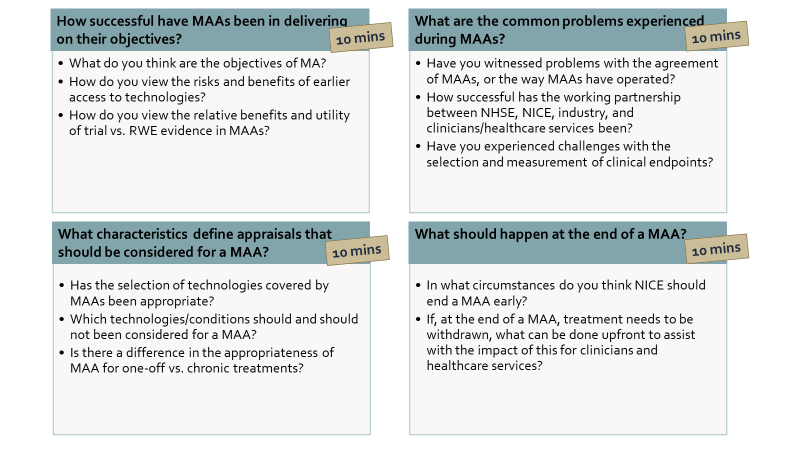


## Example quotes

The purpose of this section is to provide additional quotes to exemplify the themes presented in the manuscript. These quotes are grouped by themes accordingly. Please note that quotations have been selected to maintain the confidentiality of the participants and to avoiding sharing sensitive information discussed during the workshops.

### **Understanding the aims of MA**

- “So whether it’s been successful or not depends on what the definition of what it was set up for and what the aims and the objectives and the expectations of it was and I think each stakeholder in this had completely different expectations of what it was for, if you like” [Clinician, HST speciality]
- “In our view it’s a complicated system...There's real uncertainty as to what is the primary cause and outcomes of an MAA” [Patient Rep]
- “We kind of see the push to get quicker and faster access for everything now, ultimately, we’re going to start giving things that don’t really work” [Payer]
- “If this is trying to answer a research question and evidence question it’s then also very difficult to add in the question of managing patients…I worry that there were too many purposes together and that in trying to do so many things all at the same time you don’t achieve everything you want to” [Clinician]
- “I’d be very worried if we have a sort of uniform managed access tool which tries to …[gather] evidence about the effectiveness of the treatment and tries to act as a cost management, costs-spreading approach…it’s hard to see how those two issues do not bias each other” [Clinician]

### **Measuring the success of MA**

#### Decision-making

- "It appears it's either to delay a decision or prevent from saying no” [Patient Rep]
- “Unless we approach this almost like a formal application, and get it reviewed in terms of our final call, I just can’t see it working, and being very public about ‘if in two years these objectives are not met, then the drug will be withdrawn’” [HTA representative]
- “They come in at a high price, I think that they know there’s a psychological pressure on the committee to accept it... and withdrawing a drug is much harder than not introducing it to start with“ [HTA representative]
- “[The] example of multiple sclerosis is a good one, because nobody would want to take away those drugs now that they’re established practice. So that really calls into question the whole process of managed access if you’re not going to deal with it at the end in terms of a robust way of removing treatments available already” [HTA representative]

#### Evidence generation

- “[The] aim is to resolve uncertainty, [we] shouldn't pay for drugs where we can't resolve uncertainty” [Payer]
- “[The aim is] to allow more time for further clinical evidence to be accrued, such that a more precise cost effectiveness estimate could be produced; and, as a result, the company would then reduce their costs, if necessary, to a level that would allow it to be accepted as cost effective use” [HTA representative]
- “Even simple real-world evidence can make a big difference to how a committee approaches it and ultimately, of course, what price the company is prepared to... is prepared to pay. So I think in cancer it all works incredibly well“ [HTA representative]
- "I don't know why the scheme exists at all, really, because…nearly all the evidence we take notice of are the longer trial outcomes, and those trials would have continued anyway“ [HTA representative]
- “I think we have seen some positive correlation in terms of the outcomes collected in the real world versus what we have observed in trials. So it gives a bit of reassurance that these things do what they say on the tin” [Industry rep]
- “We had a stopping rule, and the committee were really concerned that clinicians wouldn’t adhere to the stopping rule. So we have now data generated to show, actually, they do” [Industry rep]
- "It requires us to set up data collection much too fast with a lot of difficult incentives on the edge and it’s extremely difficult to ensure and deliver very high quality data collection and that should actually be in a trial“ [Clinician]

#### Policy objectives

- “Something sort of defensible and explainable to patients who received the treatment during that period at the end of the period, so if NICE evaluates the evidence and says either we still don’t know or it’s not effective, a way to explain that to patients” [UK government official]
- “England was the first to recommend those topics [CAR T therapies] of all countries in Europe…and without managed access we wouldn’t have been able to do that” [Payer]
- “In the HST space, I think managed access has been used reactively and as a last resort to save or to avoid a negative political outcome I think for a small number of topics” [Payer]

#### Commercial objectives

- “I think what the CDF reforms have allowed …is a far more open and free conversation with companies and that freedom to talk about, look, the price you’re charging, the price you want to... charge just isn’t going to cut it but we can come to a deal... The CDF has created an opportunity to actually be flexible and find ways to get this stuff over the line when it’s important enough and I think managed access that’s one of its true successes” [Payer]
- “It’s very good for the NICE committee to have an NHS person there answering all their questions but then that NHS person is directly involved in sorting out all the commercial deals because [they] know exactly what the committee has been thinking [and the discount the company will need to offer]” [Clinician]

#### Finance

- “Not to say that they don’t still get pushed down on price and NHS England don’t still get very good deals...once something’s in the CDF we’re definitely getting cost-effective prices and actually more often than not an additional discount" [Payer]
- “There’s an enormous discrepancy between published spend on medicines at list price and what the NHS actually spends on drugs so the value of these discounts is enormous” [UK government official]
- “We could be spending on proven cost-effective treatments whereas managed access isn’t proven to be cost-effective so, arguably, you’re displacing really good value money into something that’s a gamble. So we've probably saved money versus if we paid at list price but certainly not save money in terms of net health gain” [Payer]
- “My other concern about the current system is just the amount of time and effort that’s required from those who are charged with administering it” [HTA representative]
- “It’s death by 1,000 cuts. You’re sort of paying for the uncertainty, you’re paying for the administrative burden of managed access, and paying for the data collection.” [Industry]
- “I think it was [other participant] who was saying that they’d had to pick up the costs of setting up the data collection. With cancer, fortunately, you don’t because the SACT dataset is there, but it’s this inequity, I suppose, between different disease types” [Industry]

#### Delivering early access

- “[Those technologies] wouldn’t have got over the line and that was always going to be the wrong answer for patients and for the service and for innovation and for [the] UK. And the managed access approach taken in the CDF has opened up the conversation for the system to be able to respond better than it did before." [Payer]
- “Managed access has an important part to play in our system when the drive for many years now has been to get earlier access with less and less data so we’re almost forced into managed access as part of the solution” [Payer]
- “I think without managed access we’d be in the situation where industry, for a number of breast cancer drugs, would have probably delayed submitted the drugs to NICE for some time until that further evidence was available, which obviously can be a significant amount of time, or just risk NICE rejecting the drug completely for routine access” [Patient rep]
- “We kind of see the push to get quicker and faster access for everything now, ultimately, we’re going to start giving things that don’t really work” [Payer]

#### Patient care

- “The stuff that keeps me awake is actually the HSTs because they’re... it was never guaranteed that evidence was going to be collected and then we’ve got a bunch of kids on drugs that we can’t prove are effective” [Clinician]
- "There definitely is a real frustration …and almost a desperation... they don’t really understand what the MAA’s for. They sort of do, but they don’t understand the nuances of it. They’re scared by the scoring system, and they’re incredibly worried about what happens at the end of it. So after a huge battle, and struggle, and I’m sure everybody you know, we had desperation amongst families to get on [the treatment], and then what happens at the end of the five years… it feels like, ‘Are we going to go through the same thing again?’” [Patient rep]

### **Challenges to MA**

#### Delays

- “Managing uncertainty would make more sense if a MAA kicked in more quickly, and we didn't go through a whole NICE process and then come up with an MAA…I think the Scottish system is showing us that this can happen much more quickly” [Patient rep]
- “I’m also conscious, you know, industry have a huge part to play in this. They need to engage early. Often where we’ve had problems…where things have gone into a black hole for literally months…it’s often because companies…have resisted CDF status” [Patient rep]

#### Evidence generation

- “It requires us to set up data collection much too fast with a lot of difficult incentives on the edge and it’s extremely difficult to ensure and deliver very high quality data collection and that should actually be in a trial” [Clinician]
- “The data collection agreements that are in place don’t necessarily, or aren’t necessarily, robust enough to address the key uncertainties. So they may reduce the uncertainty by waiting for trials to mature or what not. But they don’t necessarily build the business case for those QALYs“ [Industry]
- “To be in the cancer drugs fund for two or three years, really isn’t providing any useful overall survival data, which is really often what it hinges on " [HTA representative]
- “There are numerous procurement and systems in hospitals across the NHS... they all produce data in different ways… So we know that there are challenges in getting good financial data to just understand how much of a drug the NHS has actually used and at what cost, in a particular indication” [Payer]
- “With the SACT data set you don’t have... individual level patient data so to plug it into your model and there’s no comparative data so... all the committee gets is an update of trial data… and then this sort of other dataset SACT saying that actually the clinical trial data is wrong and... that puts them in a difficult position because they can’t really use that SACT data... to help them make a decision” [Payer]
- “What we are finding is often having to break bad news around managed access because we aren’t necessarily able to resolve the uncertainties all the time, they still persist and part of the reason for that is it’s not clear if the drug works.“ [Payer]
- “How do we fund stuff for rare disease post-HST MAA when we still can’t prove whether it works or not? What do we do? Do we do another managed access agreement, do we do another 10-year agreement, do we keep collecting data or, you know, at one point do we... do we say we can’t do this anymore?“ [Clinician]

#### Stakeholder engagement

- “I think transparency, clearer explanations for people to find very rapidly would be helpful” [Patient rep]
- “The anxious wait whilst people don’t know whether they’re going to be able to access the treatment in future has such a huge impact. I think even just communicating the fact that conversations are ongoing is a reassuring point” [Patient rep]
- “Patient groups don’t know that’s the system, and we’ve had incredible delays and a lot of frustration from families” [Patient rep]
- “The working partnership has improved, however more early dialogue is required to prevent delays in guidance being produced and patient access. Clearer guidance on processes and standard templates would help” [Industry rep; written submission]

#### Changes in context

- "But where there have been issues in terms of the CDF and maybe things coming out of the CDF I think they’re not failures of managed access, they’re maybe failures of... of other things. So what... one example is a drug that had its licence removed and then other examples are companies not making a submission at the end of the managed access period, which from our... from [managed single] point of view is a breach of the kind of good faith agreement that you have when you enter a managed access period as opposed to a failure of the managed access itself.” [Payer]
- "all of our MAAs came just prior to the HST change in 2017. So we never had an ICER threshold that we were working to with our original submissions. We had some rough discussions with our colleagues at NHS England and NICE but we never had any ICER guidance and then all of a sudden we’re having to look at resubmissions now and hitting ICER thresholds that were never there before... So companies like myself [and others] are being held to this ICER threshold that we never signed up to back in 2015, 2016.” [Industry rep]
- "what we’re facing for one of our CDF exits is almost a complete change in the scope. So it’s new comparators, and I mean you literally have to re-do your submission" [Industry rep]
- " a managed access agreement runs for a period of three or five years and policies change a lot in that time period, what does the landscape look like at the end of it, versus before and what are the implications for companies, patients, the NHS, with that new landscape? I’m not sure we can really answer that, and that is a real problem about what level of confidence for a company to go into a managed access agreement now” [Industry rep]

#### Contracting

- "The first two to exit are beset with problems but those problems are partly due to the fact that they were designed before we even had the Cancer Drugs Fund so pre-date some of the clearer policy lines around managed access that came with the CDF” [UK government official]
- "We are having a volume of topics exit and not one of them has been simple from my team’s perspective because companies are throwing all sorts of bizarre requests at us at the last minute and it’s delaying things in a lot of ways so we need to think about how we can tighten up that exit process." [UK government official]
- " I can think of a particular product at the moment where you know it’s probably a bit lukewarm and works in some and doesn’t work in others and then... then that feels wrong to me that you might be funding a product that perhaps isn’t working very well" [Payer]
- "When the data collection fails I think companies sometimes could blame NICE for this and it’s like where do these responsibilities lie, like who... you know, who is ultimately responsible for each component of these agreements? …the commercial data and the... like how that is collected and analysed and, yeah, and also in actually operationalising these data collections, like getting a registry fit for purpose and up and running and things like that, like is it the company, is it the registry? Sometimes it’s quite a blurred line" [Payer]
- "I think the exit process at the moment is... is very messy and it’s quite weak and so we’re getting a lot of problems when drugs are coming out, you know companies potentially refusing to submit data because they know they’re not going to get a routine recommendation, where data collection’s failed potentially and they just don’t have the data to show what... to address those clinical uncertainties. …We’ve had a few appeals as well on exit where companies aren’t happy with the... or are challenging the process of exit, for example, the scope of what’s being reviewed, so it’s meant to be a condensed review process where you’re just looking at that CDF indication and updating the same economic model with the data that’s been collected but companies are challenging that. And this is all something that we are trying to... trying to strengthen in the IMF but I think it needs to be a process that’s a bit more robust that can’t... that is less likely to be challenged so much by companies" [Payer]
- "It’s quite clear five years on, both sides probably weren’t quite aligned with what it was going to look like at the end. So we gather data and our understanding is that data that we collected would be assessed to see whether it addressed uncertainty. The reality, what’s played out, is that we’re being asked to do a whole new appraisal, and go, ‘Well that certainly wasn’t clear.’ It’s not to say one side is right or wrong, but it’s interesting how the parties come away from that with different expectations of what it may look like at the end" [Industry rep]
- "I mean I guess the difference now, there is the commercial framework that’s in place. It does very clearly lay it out" [Industry rep]
- "The exit criteria of an MAA, I understand the patient uncertainty, but from all the MAAs that we’ve done, the risk is actually on the company and if you don’t secure a positive NICE recommendation in the resubmission then the company has to continue supply. So for patients existing in the MAA, I would imagine they would have continued supply because the company has to sign-up to that as well" [Industry rep]
- "We had a very, very tough period with NHS England. We have to agree, in advance, what they call an exit strategy. So if at the end of it you don’t generate the evidence that you require when you go back to NICE in order to get your full positive recommendation, you have to clearly agree with NHS England, ‘Okay, how are you going to manage those patients at the end of it?’ They were tough, I have to say. Those discussions were so tough, at one point we just thought, ‘We have not a way forward,’ and we were just about to walk away and that was after a whole year of a number of meetings and, in the end, we pulled in [Director] in NICE, almost as an arbitrator because it was either all or nothing, and that really helped, bringing NICE into that discussion really helped and NHS England backed down a bit. But their demands, I have to say, we thought were totally inappropriate based on the discussion at the NICE committee meeting. So it was really painful. We got there in the end, but it took a whole year, and, you know, that’s another year’s delay to patient access." [Industry rep]
- "I suppose in some situations you might be able to very tightly define what outcome you need to achieve and what does that therefore mean for your price, you know, ‘If you achieve a median overall survival of X that means your price will be Y. If you don’t then it’s Z.’ I don’t know. I mean, I’m not saying that that is the way forward, but…I’ve said before, the goalposts change, I guess, by the time you exit" [Industry rep]

### **Future eligibility for MA**

- “There are different pressures on NICE, and I think the important thing is that if the managed access scheme is changed, or expanded, that the aims should be really, really clear. Because we’re working on certain assumptions, and those assumptions, in the future, might not necessarily hold” [HTA representative]
- “One, there needs to be achievable, simple evidence. So we need to have data that we know we’re going to accrue, without asking too much of the companies. Usually, that will be if another clinical trial is going to come to conclusion within the time period of the MAA. The second thing is the insistence that it is potentially cost effective” [HTA representative]
- “If we’d conducted thorough feasibility on some of…the registries we probably would have … recommended against a managed access [agreement] because they don’t have the expertise or... or the capacity to deliver these data collections” [Payer]
- “You’d only go in if you felt confident you were going to be able to generate some useful data coming out, because it is high risk and quite costly” [Industry]
- “If we only approved drugs for MAA which priced in the uncertainty, in other words had a low enough cost to be potentially… cost effective… allowing for the uncertainty and then, when we accrued further information, would allow a price increase, maybe we would get what we wanted from the MAA” [HTA representative]

Abbreviations: CDF = Cancer Drugs Fund; DoH = Department of Health; HST = Highly Specialised Technology; NHS = National Health Service, NHSE = NHS England; NICE = National Institute for Health and Care Excellence; MA = Managed access; MAA = Managed access agreement; SACT = Systemic Anti-Cancer Therapy chemotherapy dataset.
